# Cottonseed Press Cake as a Potential Diet for Industrially Farmed Black Soldier Fly Larvae Triggers Adaptations of Their Bacterial and Fungal Gut Microbiota

**DOI:** 10.3389/fmicb.2021.634503

**Published:** 2021-03-29

**Authors:** Dorothee Tegtmeier, Sabine Hurka, Patrick Klüber, Karina Brinkrolf, Philipp Heise, Andreas Vilcinskas

**Affiliations:** ^1^Department of Bioresources, Fraunhofer Institute for Molecular Biology and Applied Ecology, Giessen, Germany; ^2^Institute for Insect Biotechnology, Justus Liebig University Giessen, Giessen, Germany; ^3^Department of Bioinformatics and Systems Biology, Justus Liebig University Giessen, Giessen, Germany

**Keywords:** black soldier fly larvae, amplicon sequencing, bacteria, fungi, cottonseed press cake, core microbiome, insect development, gossypol

## Abstract

Black soldier fly larvae (*Hermetia illucens*, Diptera: Stratiomyidae) are used for the bioconversion of organic side products into valuable compounds such as proteins, lipids and chitin. However, the economic competitiveness of farmed insects compared to conventional protein production systems in agriculture and aquaculture depends on the availability of large quantities of inexpensive insect feed. Cottonseed press cake (CPC) is a side-stream of cotton production that is rich in proteins and lipids but unsuitable as feed for several farmed animals, except ruminants, due to the presence of the anti-nutritional sesquiterpenoid gossypol. Here, we tested CPC as a feed for black soldier fly larvae and studied the impact of this diet on the gut microbiome. Larvae reared on CPC developed normally and even showed a shorter life-cycle, but were smaller at the end of larval development than control larvae reared on chicken feed. The adaptability of the larvae to different diets is mediated by their versatile gut microbiome, which facilitates digestion and detoxification. We therefore used amplicon sequencing to analyze the bacterial and fungal communities associated with larvae reared on each diet, revealing differences between the larval guts and frass (residual feed substrate) as well as differences between the two diet groups. For example, *Actinomycetaceae* and Aspergillaceae were significantly enriched in guts of the CPC diet group and may help to metabolize compounds such as gossypol. Potentially probiotic yeasts and beneficial *Enterobacteriaceae*, which presumably belong to the core microbiota, were detected in high relative abundance in the gut and frass, indicating a functional role of these microbes, especially the protection against pathogens. We conclude that CPC may be suitable as an inexpensive and environmentally sustainable feed for the industrial rearing of black soldier flies.

## Introduction

The larvae of the black soldier fly (*Hermetia illucens*) are economically the most important farmed insects used for the bioconversion of organic waste into valuable compounds such as proteins, lipids and chitin (van Huis, 2013; Müller et al., 2017). These fast-growing insects can recycle large amounts of organic carbon into edible proteins and oils rather than breaking it down into CO_2_ and methane (Perednia et al., 2017). Black soldier fly larvae (BSFL) have already been approved in the EU as a feed substrate in aquaculture, and are also being considered as livestock feed.

The gut microbiota plays a key role in the adaptability of BSFL to different diets, and is regulated by a panel of antimicrobial peptides that are expressed in a diet-dependent manner (Vogel et al., 2018). Although the BSFL gut microbiome remains stable during development when larvae are reared on a uniform diet (Cifuentes et al., 2020), its composition can change significantly when the diet is varied, as shown for BSFL reared on food waste, cooked rice or calf forage (Jeon et al., 2011) and vegetable or fish meal (Bruno et al., 2019). The BSFL microbiome is shaped by diet and other biotic and abiotic factors, explaining substantial differences between the microbiomes of larvae reared in different locations (Wynants et al., 2019).

The bacterial community of BSFL has been studied in detail but the fungal community has been largely overlooked. However, the fungal community also plays an important role in digestion, nutrient supply and the breakdown of toxic metabolites in insect guts (Starmer et al., 1986; Dowd, 1992; Vega and Dowd, 2005). Nevertheless, fungi can also produce toxins (Bennett and Klich, 2003) and their numbers must therefore be controlled during the farming of insects as food and feed. Currently, there is only one comprehensive study available which evaluated the fungal microbiome of BSFL. This study has shown that also the fungal community composition was strongly dependent on the diet (Varotto Boccazzi et al., 2017).

The adaptability of the BSFL gut microbiome allows this insect to convert diverse industrial side-streams and waste products into valuable proteins. Cottonseed press cake (CPC) is an abundant industrial side-stream generated during cottonseed oil production. Its global production is about 14.7 million tons per year (Heuzé et al., 2019). Whereas most agricultural side products are rich in cellulose, pectin, lignin and other indigestible fibers, CPC is highly nutritious, comprising 27–51% protein and 8% fat as a proportion of dry matter (He et al., 2015; Kumar et al., 2015). However, cottonseeds also contain the toxic polyphenolic compound gossypol, at a concentration of 0.4–1.5% free gossypol and 2–4% bound gossypol (Pons and Eaves, 1967). This is a natural insecticide, protecting the plant from herbivores (Li et al., 2016). Accordingly, CPC is generally unsuitable as feed because it causes growth depression, infertility and disorders of digestion (Herman, 1970; Berardi and Goldblatt, 1980; Liener et al., 1990; Francis et al., 2001). Thus far, only ruminants have been shown to tolerate CPC because they produce rumen proteins that detoxify gossypol by sequestration into inactive complexes (Reiser and Fu, 1962) prior to degradation by microbes, which use the gossypol as a carbon source (Zhang et al., 2018). Reflecting this microbial activity, fermented cottonseed meal therefore has lower levels of bound and free gossypol and is enriched with vitamins and enzymes (Zhang et al., 2006, 2007; Khalaf and Meleigy, 2008; Lim et al., 2010; Mageshwaran et al., 2017).

CPC has not been tested as a feed for insect larvae, but given that the versatility of BSFL reflects the presence of an adaptable microbiome, we hypothesize that CPC would be suitable as a feed because the microbial community would adapt in order to digest and detoxify compounds such as gossypol. We therefore evaluated the impact of CPC on the development and physiological performance of BSFL and analyzed the microbial communities of the BSFL gut and frass (residual feed substrate) in populations reared on CPC compared to those reared on the standard chicken feed diet. Illumina high-throughput sequencing of the bacterial 16S rRNA gene and the fungal internal transcribed spacer (ITS) was used to characterize the microbial population in detail and to identify diet-specific differences.

## Materials and Methods

### Black Soldier Fly Breeding

Black soldier flies were obtained from Bio.S Biogas (Grimma, Germany). The flies were kept in mesh cages (Aerarium 60 × 60 × 90 cm, Bioform, Nuremberg, Germany) in a greenhouse at 25 ± 1°C, 40 ± 10% RH, and 12:12 (L:D) h according to Klüber et al. (2020) with slight modifications. Water was provided by water-soaked paper towels in a polypropylene container. Additionally, the flies were sprayed daily with water *ad libitum*. Eggs were harvested from oviposition sides (stacks of three wooded boards separated by washers) using plastic spatulas, placed in plastic boxes (19.5 × 16.5 × 9.5 cm) sprayed with water and closed with lids (200 mg eggs = ∼ 9,000 eggs per box). When ∼50% of the larvae had hatched, the lid was replaced with a fine mesh. Larvae were reared in a climate room at 27 ± 1°C and 65 ± 5% RH in the dark according to Klüber et al. (2020) with slight modifications.

As control diet we used Golddott Eierglück chicken feed (Raiffeisen, Münster, Germany). CPC (from mechanically extracted *Gossypium hirsutum* seeds) was obtained from a cotton ginning and oil mill factory (Kafantaris-Papakostas, Karditsa, Greece) where it was stored in a dry roofed warehouse for a few days to a few months until shipment. The concentration of free gossypol in the CPC constitutes about 0.06%. After delivery, the dry chicken feed and dry CPC (moisture content of 6–8%) were stored at room temperature in separate plastic lidded barrels (60 L). No transgenic feed was used in this study.

Prior to feeding to the larvae, the chicken feed and CPC were ground to an approximately similar particle size (range of 100–1,500 μm), using a Mockmill 200 grain mill (Wolfang Mock, Otzberg, Germany) and an EGK200 spice and coffee mill (Rommelsbacher, Dinkelsbühl, Germany), respectively. Particle size was measured with analytical sieves (Retsch, Haan, Germany). Freshly hatched larvae were initially fed with 10 g grinded feed. Boxes with larvae were checked daily for their need of water and feed. Additional water and feed were provided *ad libitum* until larvae reached the prepupal stage. Moisture content of the substrate was measured with a TMT-MC-7828S soil moisture meter (OCS.tec, Neuching, Germany) and adjusted to approximately 70% by spraying and mixing the substrate with water. Disposable nitrile gloves were worn throughout the experiments. Separate populations of BSFL were reared continuously on chicken feed (established in July 2018) or CPC (established in February 2019) as the only diet for several generations.

### Developmental and Physiological Parameters

Larval growth on the different diets was investigated with three replicate boxes per diet. Larvae used for the experiment were from generation 17 (chicken feed) and generation 13 (CPC). Briefly, 150 mg of eggs (same date of harvest; collected within 24 h) were placed in each box and the hatching date was recorded. Once larvae reached 3–4 mm in length (L3 larvae), pools of 50 larvae per box were weighed at 2 day intervals for all replicate boxes until 50% of the larvae had reached the prepupal stage (dark color). Subsampling was performed for each replicate box by removing larvae separately from the substrate, removing substrate particles from the larval surface with spring steel tweezers, subsequent drying with a paper tissue, and collecting 50 individuals in a weighing bowl.

The pH of the frass was measured in 2 day intervals (at the same time as larval weight measurement) by inserting pH-Fix pH 4.5–10.0 indicator strips (Macherey-Nagel, Düren, Germany). The temperature of the substrate was monitored in 2 day intervals with a TFX410 precision thermometer (Ebro, Ingolstadt, Germany). The final weight and length of 50 L5 larvae, prepupae and pupae were documented for each box. Larval length was measured for each individual under a VHX-2000 digital stereomicroscope (Keyence, Osaka, Japan) and the larval instar was determined by measuring the width of the head capsule according to Barros et al. (2019). Successful development of the stages larva–prepupa, prepupa–pupa and pupa–imago were determined in triplicates with 100 individuals each in 18.5 × 16.0 × 8.5 cm polypropylene containers. Feed was provided only for the evaluation of the development from larvae to prepupae, since prepupae and pupae do not feed. The developmental period was recorded as soon as 50% of the individuals reached the next developmental stage. Imagoes were collected within 24 h after emerging with spring steel tweezers and cold-inactivated on ice to determine their sex-specific weight and length.

Individual larval weight and gut weight were recorded 13 and 22 days after hatching. The pH of the gut was determined by placing L5 larvae on feed mixed with either 0.2% phenol red or 0.2% bromophenol blue (Bruno et al., 2019) in three replicate plastic boxes (11.5 × 9.5 × 6 cm, with holes) per diet. After incubation for 24 h, larvae were washed, and the guts were dissected under a stereomicroscope and photographed. The pH was determined by observing the coloring of the different gut sections with phenol red (pH ≥ 8.2 intensive red; pH ≤ 6.8 yellow) and bromophenol blue (pH ≤ 3.0 yellow; pH ≥ 4.6 blue). For intermediate values, a gradual color transition was observed for both indicators. Bacterial and fungal cell counts in the guts of BSFL were determined by homogenizing five guts of L5 larvae reared on each diet in a 16 ml culture tube with 5 ml phosphate-buffered saline and 1 g of glass beads (2 mm) by vortexing for 10 min. The gut homogenate was serially diluted and 200 μL of the dilutions (10^–4^–10^–8^) were plated on nutrient agar (CM0957, Oxoid, Wesel, Germany) and yeast glucose agar with chloramphenicol (Carl Roth, Karlsruhe, Germany). After incubation at 27°C for 4 days, bacterial and fungal colony forming units (CFU) on the respective agar were counted.

### Nutritional Parameters of BSFL and Feed Substrates

L5 larvae (pool of 100 g per diet) and feed (20 g per diet) were ground with a mortar under liquid nitrogen and then lyophilized for 72 h. The following analyzes relate to dry matter. 0.5 g of lyophilized larvae and 1 g of lyophilized feed were used to determine crude protein and fat. Total nitrogen was determined following the Kjeldahl method (Kjeldatherm, Vapodest 500, Gerhardt, Königswinter, Germany) to calculate the crude protein value using conversion factors <6.25 (Lenaerts et al., 2018). For crude fat determination samples were manually disintegrated for 30 min in 2 mol^⋅^L^–1^ HCl, filtered, washed neutral and dried for 2 h at 105°C. The fat was extracted automatically with n-hexane in a Soxtherm system (Gerhardt, Königswinter, Germany) and the content was determined gravimetrically.

### Sample Collection and DNA Extraction

L5 larvae from generation 12 (chicken feed) and generation 9 (CPC) were collected at the breeding facility and transferred to the laboratory. Frass samples were collected from the top left, middle and bottom right of same breeding containers and pooled. Feed samples were collected at three different time points within 1 week (days 1, 4, and 7) to identify microbes already present in the feed and to compensate for variations of the microbiota in the feed over time.

L5 larvae with a fresh weight of 100–150 mg were collected from one box per diet, washed with sterile water and rinsed twice with 70% ethanol. Separate vessels with water and ethanol were used for larvae from the different diets. Guts were dissected with sterile forceps under a stereomicroscope. Forceps were washed with sterile water and ethanol after each dissection. Single guts (nine replicates per diet for 16S rRNA gene sequencing and six replicates for ITS sequencing), 100 mg of fresh chicken feed and CPC (three replicates per diet), and 100 mg of frass (three replicates per diet) were disrupted by applying two cycles of bead beating in a FastPrep-24 (MP Biomedicals, Solon, OH, United States) for 45 s at 6.5 ms^–1^. DNA was extracted with the NucleoSpin soil kit (Macherey-Nagel) according to the manufacturer’s instructions, and checked for quantity and purity by spectrophotometry on a Take 3 plate reader (BioTek Instruments, Winooski, VT, United States).

### Analysis of the Microbial Community by Amplicon Sequencing

Libraries were prepared and sequenced by LGC Genomics (Berlin, Germany) using primers U341F (5′-CCT AYG GGR BGC ASC AG-3′) and U806R (5′-GGA CTA CNN GGG TAT CTA AT-3′) (Sundberg et al., 2013) to amplify variable region V3–V4 of the 16S rRNA gene of bacteria and archaea, or primers fITS7 (5′-GTG ART CAT CGA ATC TTT G-3′) (Ihrmark et al., 2012) and ITS4 (5′-TCC TCC GCT TAT TGA TAT GC-3′) (White et al., 1990) to amplify the fungal ITS2 region. The libraries were sequenced on an Illumina MiSeq V3 (San Diego, CA, United States) to generate ∼20,000 paired-end reads per sample with a read length of 300 bp. Samples were multiplexed and pooled for sequencing.

Demultiplexing of samples and the clipping of adapters and primers were carried out by LGC Genomics using bcl2fastq 2.17.1.14 software (Illumina, Inc., San Diego, CA, United States). Reads were analyzed using QIIME 2020.6 (Bolyen et al., 2019). ITS sequences were also trimmed if the synthesized strand reached the second sequencing adapter (read-through) using the cutadapt plugin (Martin, 2011) before further analysis. Only forward reads of the ITS amplicons were analyzed. We used the DADA2 plugin (Callahan et al., 2016) for error correction, quality control, filtering of chimeric sequences, and the creation of an amplicon sequence variant (ASV) table containing the number of sequences for each observed ASV per sample.

Bacterial taxonomic classification was carried out using a self-trained naïve Bayes classifier on SILVA 132 QIIME-compatible release with 99% sequence identity (Quast et al., 2013). We also trimmed the reference sequences to the specific region of the 16S rRNA gene targeted with the amplicon primers (Werner et al., 2012). Confidence was set to 0.7 as recommended (Bokulich et al., 2018). Fungal taxonomic classification was carried out using a self-trained naïve Bayes classifier on UNITE 8.2 QIIME-compatible release with 99% sequence identity (Abarenkov et al., 2020) and a minimum confidence of 0.94 (Bokulich et al., 2018). Sequences originating from mitochondria or chloroplasts were removed from the classified datasets. Alpha diversity was based on Faith’s phylogenetic diversity (Faith, 1992) and observed ASVs. Beta diversity was determined using UniFrac distance metrics (Lozupone and Knight, 2005), and we rarefied the 16S rRNA and ITS data to equal sequencing depths of 5,484 and 2,639 reads, respectively.

### Statistical Analysis

The linear relationship between weight gain and pH of the frass was calculated using Pearson product-moment correlation (Hilgers et al., 2019). Significant differences in developmental and physiological parameters were tested with Student’s *t*-test. Differences in relative abundances of certain taxa between the two diet groups were determined by applying Student’s or Welch’s *t*-test (Welch, 1947) depending on the homogeneity of variance verified by a Levene’s test (Levene, 1960) with significance at 0.05. For statistics and graphics we used Microsoft Excel and R v4.0.3 with packages qiime2R, tidyverse, car, dplyr, plyr, scales, ggpubr, and ggplot2.

## Results

### Black Soldier Fly Developmental, Physiological, and Nutritional Parameters

The life cycle of black soldier flies reared on CPC showed many similarities to that of the flies raised on chicken feed, but the total developmental cycle was significantly (*p* < 0.001; df = 4; *t* = 2.13) shorter on the CPC diet (43.4 ± 1.8 days) compared to the chicken feed diet (47.3 ± 3.5 days). Hatching time, larval development and the transition from prepupa to pupa were similar on both diets, whereas intrapuparial metamorphosis was significantly (*p* < 0.001; df = 4; *t* = 2.13) faster in the CPC diet group. The rates of successful development (larva–prepupa and pupa–imago) were also similar for both diets. Only the developmental success from prepupa to pupa was significantly (*p* < 0.000005; df = 4; *t* = 2.13) lower on the chicken feed diet (90.7%), whereas 100% of the prepupae in the CPC diet group reached the pupal stage (Table 1).

**TABLE 1:** Developmental and physiological parameters of black soldier flies reared on chicken feed (CF) and cottonseed press cake (CPC).

	**Sample size**	**CF**	**CPC**
Hatching time (d)	≥50%	3.0 ± 0.8	3.0 ± 0.8
Larval development (d)^a^	≥50%	22.3 ± 0.5	23.7 ± 0.5
Prepupa–pupa (d)	*n* = 300	11.3 ± 1.7	10.0 ± 0.0
Intrapuparial metamorphosis (d)^b^	*n* = 300	10.7 ± 0.5	6.7 ± 0.5
Total development (d)^c^	*n* = 300	47.3 ± 3.5	43.4 ± 1.8
Successful development larva–prepupa (%)	*n* = 300	100.00 ± 0.00	99.33 ± 0.94
Successful development prepupa–pupa (%)	*n* = 300	90.67 ± 0.47	100.00 ± 0.00
Successful development pupa–imago (%)	*n* = 300	97.00 ± 2.45	99.67 ± 0.47
Final larval weight (mg)	*n* = 300	285.14 ± 26.40	160.36 ± 16.08
Final larval length (mm)	*n* = 150	25.96 ± 0.07	18.65 ± 0.18
Weight prepupa (mg)	*n* = 150	219.60 ± 18.69	101.80 ± 9.59
Length prepupa (mm)	*n* = 150	23.43 ± 0.12	16.82 ± 0.40
Weight pupa (mg)	*n* = 150	168.97 ± 10.74	93.89 ± 4.91
Length pupa (mm)	*n* = 150	22.65 ± 0.08	16.95 ± 0.17
Weight imago ♂ (mg)	*n* = 90	89.42 ± 8.51	40.18 ± 3.98
Weight imago ♀ (mg)	*n* = 150	103.57 ± 8.29	59.09 ± 3.56
Weight imago, total (mg)	*n* = 255	98.05 ± 8.02	50.64 ± 3.50
Length imago ♂ (mm)^d^	*n* = 90	16.76 ± 0.25	14.41 ± 0.19
Length imago ♀ (mm)^d^	*n* = 150	17.50 ± 0.25	15.77 ± 0.24
Length imago, total (mm)^d^	*n* = 255	17.20 ± 0.24	15.15 ± 0.23
Weight 13 d larvae (mg)	*n* = 25	124.17 ± 19.49	118.87 ± 9.66
Weight 22 d larvae (mg)	*n* = 25	247.00 ± 38.51	162.36 ± 25.65
Gut weight 13 d larvae (mg)	*n* = 25	25.43 ± 7.75	35.73 ± 5.01
Proportion of gut to total body of 13 d larvae (%)	*n* = 25	20.48 ± 6.24	30.06 ± 4.21
Gut weight 22 d larvae (mg)	*n* = 25	42.70 ± 19.50	52.14 ± 13.78
Proportion of gut to total body of 22 d larvae (%)	*n* = 25	17.29 ± 7.89	32.11 ± 8.49
pH anterior midgut	*n* = 10	≤6.8	≤6.8
pH middle midgut	*n* = 10	≤3.0	≤3.0
pH posterior midgut	*n* = 10	≥8.2	≥8.2
pH hindgut	*n* = 10	≥6.8	≥6.8
Crude protein of larvae (%DM)	*n* = 300	38.60 ± 1.24	47.43 ± 0.08
Crude protein of feed (%DM)	–	18.47 ± 0.18	24.76 ± 0.62
Crude fat of larvae (%DM)	*n* = 300	33.94 ± 1.09	23.34 ± 1.23
Crude fat of feed (%DM)	–	2.49 ± 0.03	8.65 ± 0.51

*Values are means ± standard deviation.*

*^a^Period from hatching to ≥50% prepupal populations.*

*^b^Period until ≥50% of the adults emerged from the pupae.*

*^c^Period from oviposition to adults emerging.*

*^d^Distance between cranial antennae attachment points and abdominal appendages.*

**FIGURE 1:**
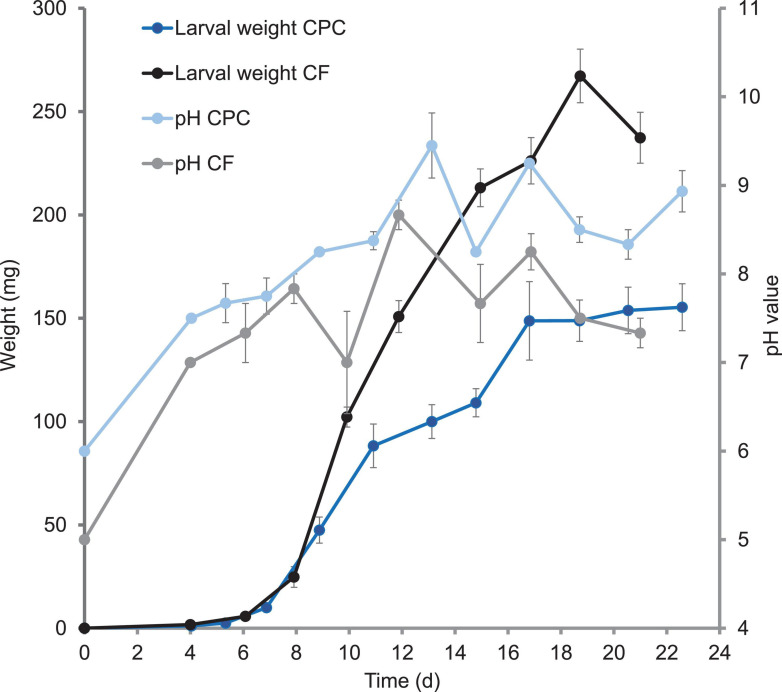
Growth curve of larvae reared on chicken feed (CF) and cottonseed press cake (CPC), and the pH of the corresponding frass. The average body weight per BSFL and average pH values of the frass are shown. Symbols are means (+ SEM) of measurements with three replicates (containers with BSFL + substrate).

We also observed major differences in larval growth between the two diets. Weight gain was similar for the first 11 days on both diets. However, whereas larvae reared on chicken feed gained weight continuously until day 19, those reared on CPC gained little weight from days 11 to 15, then experienced a 2 day growth phase followed by a longer plateau lasting 5.5 days until ≥50% prepupae were present (Figure 1). A pause in larval growth was also observed in a repeating trial only with larvae reared on CPC but not with larvae reared on chicken feed (Supplementary Figure 1). The final larval weight was significantly (*p* < 0.00001; df = 10; *t* = 1.83) lower in the CPC diet group and the weight of imagoes reared on CPC was lower than that of imagoes reared on chicken feed. In contrast, the average gut weight of larvae reared on CPC was significantly higher (*p* < 0.00001 after 13 days and *p* < 0.03 after 22 days; df = 43; *t* = 1.68) than corresponding samples in the chicken feed diet group. Therefore, the proportion of the gut to the total larval body was also higher for larvae reared on CPC compared to larvae reared on chicken feed after 13 days and after 22 days (Table 1).

The pH of the frass showed a similar trend during larval development on both diets, but the pH of the CPC frass was slightly higher than that of the chicken feed frass. Furthermore, we observed a moderate negative correlation between growth and pH from days 9 to 15 (*r* = –0.47) only with larvae reared on CPC. The increase in pH of the frass goes along with the slowdown of growth on days 11–15, followed by a decrease in pH accompanied by the resuming of larval growth (Figure 1). The temperature of the substrate mixed with larvae was similar in each replicate box. Furthermore, there were only minor differences in temperature between the boxes with larvae reared on chicken feed (30.8 ± 2.36°C) and the boxes with larvae reared on CPC (29.5 ± 0.84°C) throughout the period of measurement (Supplementary Table 1). The luminal pH of the various gut sections was similar for larvae reared on both diets. The lumen of the anterior midgut was slightly acidic (pH ≤ 6.8). The lumen of the middle midgut contained a strongly acidic region (pH ≤ 3.0), whereas the lumen of the posterior midgut was alkaline (pH ≥ 8.2). The lumen of the hindgut was neutral to alkaline (pH ≥ 6.8) given that some individuals showed orange coloring (pH 6.8–8.2) and some individuals showed intensive red coloring of the hindgut (pH ≥ 8.2) when incubated with phenol red, regardless of diet (Supplementary Figure 2).

The count of bacterial CFU was 2.6 × 10^7^ mg^–1^ gut in BSFL reared on CPC and 6.8 × 10^6^ mg^–1^ gut in BSFL reared on chicken feed. The count of fungal CFU was much lower than the count of bacterial CFU, with 5.5 × 10^4^ mg^–1^ gut and 5.0 × 10^5^ mg^–1^ gut in BSFL reared on CPC and chicken feed, respectively. Crude protein of the larvae reared on CPC was higher than that of larvae reared on chicken feed whereas for crude fat the opposite was the case (Table 1).

### Analysis of the Bacterial and Archaeal Communities by Amplicon Sequencing

**FIGURE 2:**
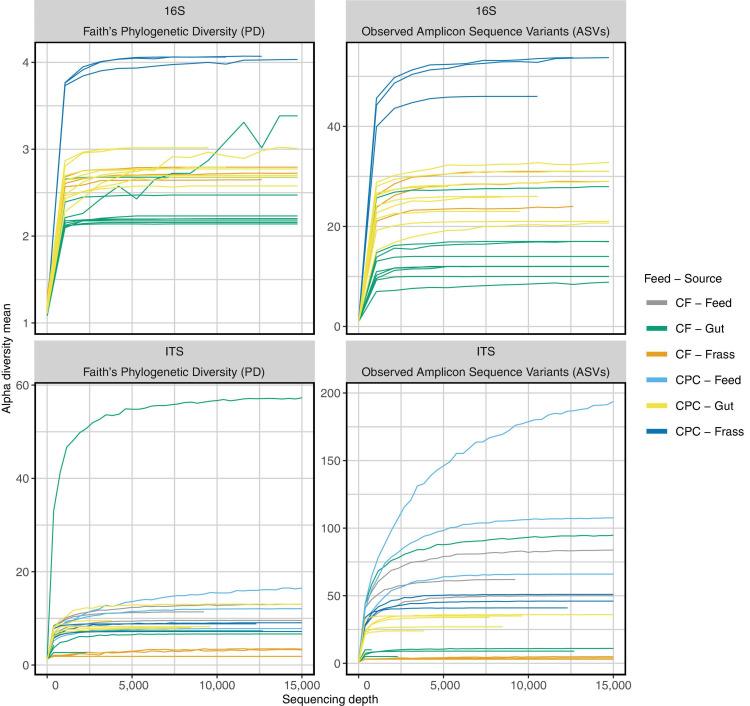
Rarefaction curves of 16S rRNA gene and ITS sequencing for all samples. Curves reach a plateau for nearly all samples of the chicken feed (CF) and cottonseed press cake (CPC) diet groups after ∼10,000 reads. Even samples with fewer reads reach a plateau for ITS and 16S rRNA gene sequencing. Therefore, higher sequencing depth does not influence microbial diversity (Faith’s phylogenetic diversity and observed amplicon sequence variants) found in the sequencing data.

We generated 711,390 read pairs for the 30 samples (nine samples of individual guts, three feed samples, and three frass samples per diet). Quality control, including the removal of chimeric sequences, reduced the total to 605,424 read pairs. After joining forward and reverse reads, the average read length was 419 bp. Thereby, the numbers of reads per sample ranged from 5,484 to 40,144. Rarefaction curves suggested the sequencing depth was sufficient (Figure 2).

After using DADA2 and our self-trained naïve Bayes classifier based on SILVA 132 followed by removing mitochondrial and chloroplast sequences we identified 170 unique amplicon sequence variants (ASVs) across our 30 samples. The same classifier assigned taxonomic labels down to the genus level for 146 of the 170 ASVs. All classified ASVs belonged to the domain Bacteria; no Archaea were found. The detailed results of the classification on all taxonomic levels (from phylum to species) and relative abundances of ASV counts are shown in Supplementary Table 2.

**FIGURE 3:**
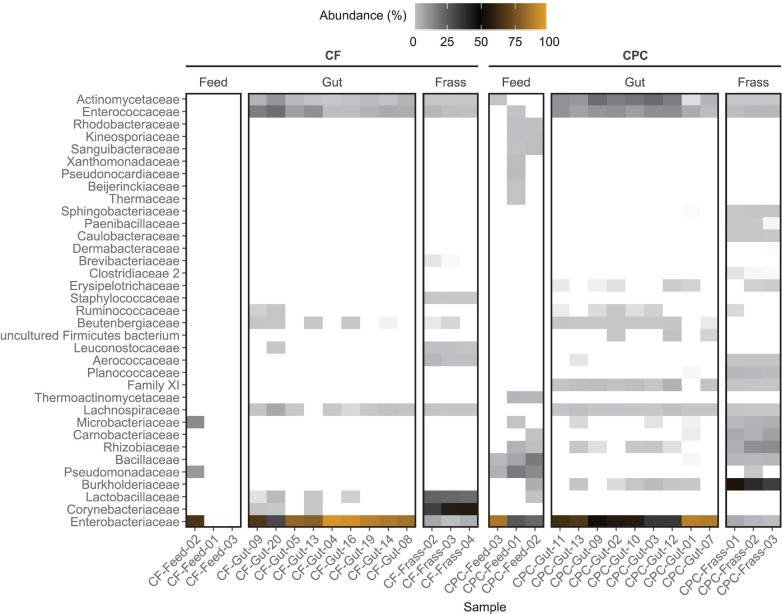
Heat map showing the family-level composition of the bacterial community of feed, BSFL guts and BSFL frass in the chicken feed (CF) and cottonseed press cake (CPC) diet groups based on 16S rRNA gene amplicon sequencing. The relative abundances of amplicon sequence variant (ASV) counts are collapsed to the family level for all replicates. Undefined families are not shown. Percentages of relative abundances are displayed by coloring from light gray over black to orange representing the highest abundance. Families depicted in white were not detected in the sample. Only the top 35 families are shown, measured by the mean counts per family level for all samples.

For all feed samples, the sum of ASV counts was very low (maximum 428 counts per sample). No bacteria were found in two of the three chicken feed samples, and only 21 counts were detected in the remaining sample. These were classified as *Enterobacteriaceae* (*Pantoea*), *Pseudomonadaceae* (*Pseudomonas*), and *Microbacteriaceae* (*Curtobacterium*). In contrast, CPC feed samples featured a more diverse bacterial community dominated by different groups of *Enterobacteriaceae, Pseudomonadaceae*, and *Bacillaceae*, which were present in all replicate feed samples (Figure 3). *Enterobacteriaceae* were the dominant family in all gut samples. However, the relative abundance was significantly (*p* < 0.05) higher in guts of larvae reared on chicken feed. Furthermore, *Enterococcaceae* and *Actinomycetaceae* were present in all replicate gut samples from both diet groups. Interestingly, *Actinomycetaceae* were significantly (*p* < 0.002) enriched only in the guts of larvae reared on CPC. *Lachnospiraceae* were present at a low abundance in almost all gut samples, regardless of the diet. *Burkholderiaceae*, *Rhizobiaceae*, *Erysipelotrichaceae*, and *Clostridiales* Family XI were only found in gut samples of larvae reared on CPC (at least five of nine replicates) whereas *Corynebacteriaceae* and *Lactobacillaceae* were only present in gut samples of larvae reared on chicken feed (at least three of nine replicates) (Figure 3).

We observed pronounced differences between the two diet groups at the genus level. *Morganella* was the most prominent genus (67.2–95.6% in eight of nine replicates) in the guts of larvae reared on chicken feed, whereas *Providencia* was more abundant in the CPC diet group (≥32% in seven of nine replicates, compared to ≤2% in all replicates in the chicken feed group) (Supplementary Table 2 and Supplementary Figure 3). Almost all bacterial families found in gut samples were also present in frass samples, although the relative abundance varied. However, we also observed pronounced differences between the gut and frass microbiomes, especially in the CPC group where families such as *Sphingobacteriaceae*, *Paenibacillaceae*, *Clostridiaceae* 2, and *Caulobacteraceae* were found in the frass but were completely absent from the gut. CPC frass was dominated by *Burkholderiaceae* (mostly classified as *Alcaligenes*, 44–54%), *Rhizobiaceae* (7–18%), *Carnobacteriaceae* (7–11%), *Microbacteriaceae* (4–7%), and *Bacillaceae* (4–5%) in all replicates, whereas chicken feed frass was dominated by *Corynebacteriaceae* (44–57%), *Lactobacillaceae* (30–33%), and *Enterobacteriaceae* (3–9%). Furthermore, *Staphylococcaceae* and *Aerococcaceae* were present at a lower abundance in chicken feed frass but were completely absent from the gut samples. The most abundant families in the CPC frass were also present in some replicates of the feed samples, but were absent or scarce in the corresponding gut samples (Figure 3 and Supplementary Table 2).

**FIGURE 4:**
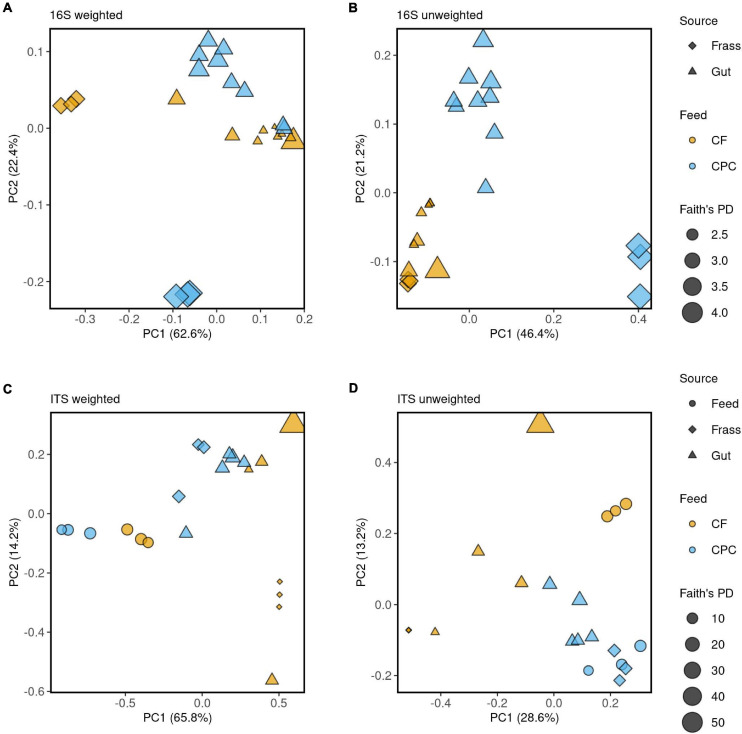
Principal coordinates analysis (PCoA) of the microbial community structure among chicken feed (CF) and cottonseed press cake (CPC), BSFL guts and BSFL frass samples. **(A,B)** PCoA of bacterial communities calculated on weighted and unweighted UniFrac distance metrics, respectively. **(C,D)** PCoA of fungal communities calculated on weighted and unweighted UniFrac distance metrics, respectively. The 16S rRNA and ITS data were rarefied to an equal sequencing depth 5,484 and 2,639, respectively. The source of the sample is indicated by symbols color coded by feed source (CF and CPC), as displayed in the legend on the right side of the figure. Faith’s phylogenetic diversity (PD) is indicated by the size of the symbol.

When larvae were reared on CPC, most of the gut and frass samples showed a higher alpha diversity of bacteria (Faith’s phylogenetic diversity and observed ASVs) when compared to those raised on chicken feed (Figure 2 upper part; Figures 4A,B). Principal coordinate analysis (PCoA) of weighted UniFrac distances (Figure 4A) revealed that some gut samples of larvae reared on both diets clustered together, whereas PCoA of unweighted UniFrac distances (Figure 4B) indicated more differences between the two diet groups. Little similarity was observed between gut samples and frass samples on either diet (Figure 4A), but the differences were greater for the CPC diet group. We also observed a large difference between CPC and chicken feed frass samples (Figures 4A,B).

### Analysis of the Fungal Community by Amplicon Sequencing

We generated 535,559 raw reads for ITS sequencing from the 24 samples (six samples of individual guts, three feed samples, and three frass samples per diet), with 488,426 reads remaining after quality control (540–99,232 per sample). After trimming, the read length range was 83–273 bp. The average read length was 259 bp. Rarefaction curves suggested the sequencing depth was sufficient (Figure 2). DADA2 identified 854 unique ASVs across 24 samples, 413 of which were classified to the genus level using our naïve Bayes classifier based on UNITE 8.2 with a minimum confidence of 0.94. The detailed results of the classification on all taxonomic levels (from phylum to species) and relative abundances of ASV counts are shown in Supplementary Table 3.

**FIGURE 5:**
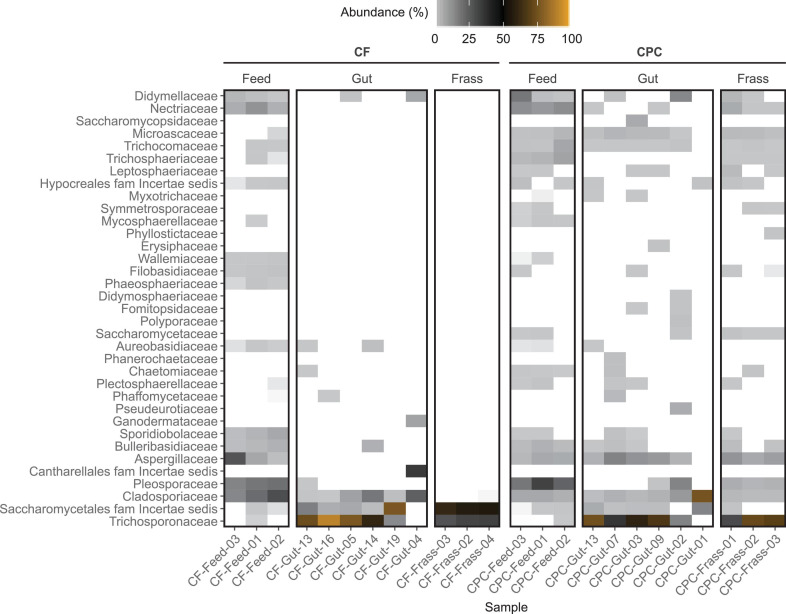
Heat map showing the family-level composition of the fungal community of feed, BSFL guts and BSFL frass in the chicken feed (CF) and cottonseed press cake (CPC) diet groups based on ITS amplicon sequencing. The relative abundances of amplicon sequence variant (ASV) counts are collapsed to the family level for all replicates. Undefined families are not shown. Percentages of relative abundances are displayed by coloring from light gray over black to orange representing the highest abundance. Families depicted in white were not detected in the sample. Only the top 35 families are shown, measured by the mean counts per family level for all samples.

The ITS amplicon sequencing revealed higher sums of ASV counts in the feed samples (9,579–99,232 counts per sample) compared to the 16S amplicons. Most of the classified ASVs in chicken feed samples represented the families Cladosporiaceae (all *Cladosporium*), Pleosporaceae (*Alternaria* and *Stemphylium*), Aspergillaceae (mostly *Xeromyces* and *Aspergillus*), and Nectriaceae (mostly *Fusarium*) and were found in all replicates. These families were also found in all replicates of the CPC feed samples but their relative abundance was on average lower (Figure 5 and Supplementary Figure 4). However, most of the ASV counts in the CPC feed samples (38–68%) could not be classified, whereas only a minority of sequences in the chicken feed samples (1–11%) remained unclassified.

In all gut and frass samples (regardless of diet) less than 21% of the ASV counts remained unclassified (Supplementary Table 3). The fungal gut microbiota of larvae reared on chicken feed and CPC was dominated by the family Trichosporonaceae (49.6–90.4% in four samples of larvae reared on chicken feed and five samples in larvae reared on CPC). Furthermore, the family Cladosporiaceae (all *Cladosporium cladosporioides*) and Saccharomycetales family *incertae sedis* (all *Diutina rugosa*) were relatively abundant in at least five replicates of both diets. *Trichosporon* (all classified as *Trichosporon asahii*) and *Diutina* (all classified as *Diutina rugosa*) were the only two genera found in frass samples of larvae reared on chicken feed, whereas frass samples of larvae reared on CPC showed a highly diverse community of many different fungal groups (Figure 5 and Supplementary Table 3).

Although the relative abundance of the Aspergillaceae was higher in the feed samples of both diets (2.5–38.8%), this family was only present in the guts of larvae reared on CPC (5.5–17.9% in five of six replicates) and was completely absent from the guts of larvae reared on chicken feed (Figure 5). Most Aspergillaceae in the gut were assigned to the genus *Aspergillus*. Most of them were not classified to species level, except *Aspergillus glaucus*, *A. penicillioides*, and *A. protuberus.* The remaining Aspergillaceae were mostly classified as *Xeromyces bisporus*, which showed a higher relative abundance (1–12%) in four of six samples of larvae fed on CPC. Furthermore, Trichocomaceae and Microascaceae (all classified as *Acaulium acremonium*), which were present in most of the replicates of larvae reared on CPC, were not detected in any replicates of larvae reared on chicken feed (Figure 5 and Supplementary Table 3).

When larvae were reared on CPC, most gut and frass samples showed a higher alpha diversity of fungi (Faith’s phylogenetic diversity and observed ASVs) compared to chicken feed (Figure 2 lower part; Figures 4C,D). In contrast to the bacterial microbiome, there was more similarity between the fungal communities in the gut and frass samples of larvae reared on CPC (Figures 4C,D). Few similarities between gut samples of larvae reared on the different diets were observed in the PCoA of unweighted UniFrac distances, whereas the PCoA of weighted UniFrac distances showed that some gut samples representing the two diet groups clustered closer together. Like the bacterial microbiome, the fungal community in the CPC frass differed substantially from that in the chicken feed frass (Figures 4C,D).

## Discussion

### Developmental, Physiological, and Nutritional Parameters of Black Soldier Flies

Here, we show that BSFL can be reared on CPC despite the presence of the normally insecticidal secondary metabolite gossypol. This is corroborated by the significantly shorter developmental time and the higher rates of successful development of prepupa to pupa of BSF reared on CPC. In contrast, several other insects show lower rates of successful development and a prolonged developmental time when subjected to gossypol (Bottger and Patana, 1966; Meisner et al., 1978; Stipanovic et al., 2006). The significantly shorter intrapuparial metamorphosis might be related to differences in microbial community composition in BSF reared on CPC, since microbial symbionts can produce hormones that influence insect signaling pathways to promote development and metamorphosis of the host (Paludo et al., 2018; Hammer and Moran, 2019; Wu et al., 2020). The accelerated development and higher rate of successful development of BSFL fed on CPC observed in this small-scale BSF-breeding facility may also apply to industrial insect farming and might be profitable for large-scale production.

The similar early growth performance of BSFL reared on CPC in comparison to the control group reared on chicken feed is remarkable, indicating that freshly-hatched larvae rapidly convert the substrate and are not harmed by the toxic components of CPC during this time period. However, the subsequent slowdown of larval growth for several days contributed to a significantly lower final weight. In the Lepidoptera gossypol also caused a lower weight of larvae and pupae (Bottger and Patana, 1966; Stipanovic et al., 2006) which is attributed to an inhibition of digestive enzymes (Meisner et al., 1978). However, such a negative effect on the insect’s digestion would also cause a significant delay in development and a decrease in rates of successful development as also shown by Meisner et al. (1978), which is not the case for BSFL. The brief pause in growth observed in the CPC diet group after 11 days may be caused by other effects of gossypol e.g., by an increased energy-consuming detoxification activity due to an accumulation of this compound at this time point. However, so far we do not know the exact concentration of gossypol of this particular CPC. According to the manufacturer the concentration of free gossypol in the CPC constitute about 0.06% but can vary depending on humidity conditions of the cotton when harvested. Since most of the gossypol in the seeds is bound to proteins (2–4% bound gossypol; Pons and Eaves, 1967), we assume that the amount of bound gossypol is also higher than the amount of free gossypol in the CPC used in this study.

The high protein and fat content of CPC suggests that interruption of growth and the lower final larval weight of BSFL reared on CPC are likely not a result of malnutrition. However, such a growth pause or a period of low weight gain would be unfavorable for large-scale production. Since BSFL reared on CPC showed highest weight gain in the period from days 7 to 11, an early harvest of the larvae when industrially reared should be considered. Although the CPC feed showed a much higher fat content as chicken feed, larvae fed with CPC even showed a lower fat content than larvae fed with chicken feed. The low fat and high protein content of these larvae might also be beneficial for future food and feed use. However, detailed measurements, including the quantification of gossypol in the feed, larvae and frass at different time points, as well as a more detailed evaluation of the nutritional value of the CPC and the larvae, would be required to confirm our hypotheses.

Growth inhibition might also be related to the increase in pH, implied by the negative correlation between growth and pH from days 9 to 15 for the larvae reared on CPC. The increase in pH during the rearing on nitrogen-rich substrates is caused by the formation of ammonia (Green and Popa, 2012; Klammsteiner et al., 2020; Pang et al., 2020; Parodi et al., 2020), which can also inhibit gowth of dipteran larvae (Wang and Leung, 2015; Dias et al., 2019). However, also other so far unknown effects, e.g., other incompatible components of the CPC diet may cause a pause of larval growth.

The pH of the BSFL gut showed a remarkable transition from the acidic region (≤3.0) in the middle midgut to the alkaline region (≥8.2) in the posterior midgut, as previously reported (Bruno et al., 2019). Although we and others (Klammsteiner et al., 2020) found that the type of diet influences the pH of the substrate in which the larvae grow, the pH of the gut lumen appears to be largely unaffected by the diet and instead reflects the specific morphofunctional features of the dipteran midgut. Like the BSFL, other dipteran larvae such as those of the housefly (*Musca domestica*) and the fruit fly (*Drosophila melanogaster*) feature an acidic region (≤3.0) in the middle midgut (Lemos and Terra, 1991). The low pH is generated by specialized midgut cells, so-called copper cells (McNulty et al., 2001; Dubreuil, 2004; Shanbhag and Tripathi, 2009), which were recently also found in BSFL (Bonelli et al., 2019). Our data suggest that the luminal gut pH is host-specific and is not strongly affected by diet-dependent shifts in the microbiome. However, the luminal pH of the insect gut probably influences the colonization of the gut by certain microbes based on their functional pH range. Populations of *Salmonella enterica* and *Escherichia coli* in chicken manure, were reduced by BSFL which is attributed to changes in pH of the manure (Erickson et al., 2004). Furthermore, passage through the acidic and alkaline regions of the gut leads to the selection of certain microorganisms which colonize the gut and also the substrate.

The significantly higher gut weight of larvae reared on CPC might be caused by a higher feed intake and a higher microbial colonization density, in order to boost the metabolic activity (e.g., gossypol degradation) within the gut. Studies with germ-free cockroaches have also shown that individuals raised under aseptic conditions showed a significantly lower gut weight when compared to normal cockroaches with an abundant gut microbiota (D. Tegtmeier, unpublished data). This suggests that the microbial load probably also affects the gut weight of BSFL. The higher gut weight of larvae reared on CPC is further corroborated by the higher bacterial CFU counts in the guts of these larvae. However, these numbers only represent the proportion of BSFL gut bacteria from both diet groups, that can be cultured on standard medium, while the total quantity is likely higher.

### The BSFL Bacterial and Archaeal Communities

The presence of *Enterobacteriaceae* (mostly *Morganella* and *Providencia*), *Enterococcaceae*, and *Actinomycetaceae* in all individual gut samples regardless of diet indicates that these groups belong to the BSFL core microbiome. The dominance of *Morganella* in gut samples from larvae in the chicken feed group and in most corresponding samples from the CPC group agrees with studies by Wynants et al. (2019), who also found a dominance of this genus in BSFL reared at two different locations. Furthermore, *Morganella* was prominent in BSFL reared on chicken feed (Cifuentes et al., 2020) and calf forage, and it was also present (albeit with lower relative abundance) when larvae were fed other diets (Jeon et al., 2011). Besides *Morganella*, also the genera *Providencia* and *Enterococcus* (*Enterococcaceae*) were previously found to be abundant in BSFL guts regardless of diet, rearing conditions or developmental stage, suggesting they form the core microbiome (Zheng et al., 2013; Wynants et al., 2019; Cifuentes et al., 2020). Also, a cultivation-dependent study has shown that the majority bacteria cultivated from BSFL guts belong to the *Enterobacteriaceae* and a large proportion of the isolates were identified as *Providencia*, *Morganella*, and *Enterococcus* (Callegari et al., 2020). *Providencia* may even be vertically transmitted, because it was detected in imagoes and eggs (Zheng et al., 2013). This is a reasonable assumption because *Providencia* and *Enterococcaceae* found in the guts samples in our experiments were completely absent in feed samples and may therefore be vertically transmitted via the eggs or taken up by the larvae from the surface of the eggs.

The high relative abundance of *Enterobacteriaceae* (in particular *Morganella* and *Providencia*) indicates a functional role of these bacteria within the BSFL gut. *Providencia rettgeri* and *Morganella morganii* are also abundant endogenous members of the gut microbiome of the burying beetle (*Nicrophorus vespilloides*), both in adults (Vogel et al., 2017; Heise et al., 2019) and in larvae (Vogel et al., 2017; Wang and Rozen, 2017), and were reported to protect larvae against pathogens (Wang and Rozen, 2018). Furthermore, *P. rettgeri* and *M. morganii* produce extracellular bacteriolytic enzymes that can degrade components of *E. coli* and *Pseudomonas aeruginosa* cell walls (Branca et al., 1996; Janda and Abbott, 2015). The high relative abundance of *Morganella* and *Providencia* in L5 larvae may explain why we did not detect *Pseudomonas* in any of the gut samples, although it showed a high relative abundance in chicken feed and CPC samples. This may also explain our frequent observation of *Pseudomonas* contamination, revealed by a green biofilm on top of the substrate and a characteristic odor of linden blossom only present during the initial rearing phase (2–6 days). This contaminant is usually eliminated by the larvae when they gain weight and reach a certain size. Furthermore, *Pseudomonas* was absent in BSFL samples from rearing locations with a high relative abundance of *Morganella*, but was present in samples where *Morganella* was scarce (Wynants et al., 2019). The reanalysis of published data (Cifuentes et al., 2020), revealed a negative correlation between the occurrence of *Pseudomonas* and *Morganella* + *Providencia* (*r* = −0.26) suggesting that these two genera help BSFL to resist colonization by pathogens.

*Morganella* and possibly other *Enterobacteriaceae* in BSFL guts likely contribute to the digestion of the protein-rich substrate and the breakdown of uric acid, as *Morganella morganii* possesses genes for proteases (Chen et al., 2012; Zamaliutdinova et al., 2014) and exhibits urease activity (Janda and Abbott, 2015). Furthermore, *Morganella*, and other *Enterobacteriaceae* are known for oxidative deamination of various amino acids and nitrate reduction (Janda and Abbott, 2015). In the course of nitrogen metabolism several *Enterobacteriaceae* (including *M. morganii*) generate ammonia (Özoğul, 2004). Therefore, ammonia emissions during the BSFL rearing process, are attributed (at least partially) to the abundant genera *Morganella* and *Providencia* in the guts of BSFL.

Most of the bacteria detected in the feed were not found to be abundant in gut samples of L5 larvae, suggesting the feed is unlikely to be the major source of inoculum for establishing the bacterial community in the gut. However, we found that some groups present in the CPC feed were also present in the frass. The abundance of certain bacterial groups only in the feed and frass but not in the gut indicates that many ingested bacteria find the physiochemical conditions in the gut unwelcoming (especially the extreme pH conditions) or are displaced by other microbes. This observation also explains the pronounced differences between the bacterial communities of the frass and the gut (Jiang et al., 2019; Wynants et al., 2019; Cifuentes et al., 2020).

The higher bacterial alpha diversity in BSFL reared on CPC may reflect the greater complexity of the diet, as previously shown for BSFL (Jeon et al., 2011) and other insects (Huang et al., 2013). In contrast to our results, most previous studies of the BSFL microbiome did not identify *Actinomycetaceae* as an abundant component, despite the use of diverse substrates including the Gainesville diet, different types of food waste, vegetables, and fish meal (Zheng et al., 2013; Bruno et al., 2019; Jiang et al., 2019; Wynants et al., 2019). *Actinomycetaceae* have been reported as part of the BSFL gut microbiome when larvae are reared on chicken feed, albeit with a low abundance (Cifuentes et al., 2020) similar to our results obtained with BSFL reared on chicken feed. In our study, the relative abundance of *Actinomycetaceae* was significantly higher in the larvae reared on CPC, suggesting that this bacterial family may play a key role in the metabolism of CPC-specific components such as gossypol. *Actinomycetales* are known for the degradation of complex molecules such as lignocellulose (McCarthy, 1987; Wang et al., 2014) and a variety of pesticides including many phenolic compounds (Schrijver and Mot, 1999). Gossypol is a phenolic pesticide (Li et al., 2016) which supports the potential role of these bacteria in its degradation. Other families present only in larvae reared on CPC (*Burkholderiaceae*, *Rhizobiaceae*, *Erysipelotrichaceae*, and *Clostridiales* Family XI) may also be involved in the degradation of such compounds, but little is known about gossypol degradation in bacteria. The only gossypol-degrading bacterial isolate known thus far is a *Bacillus subtilis* strain isolated from the cow rumen, but this is not yet available in a culture collection (Zhang et al., 2018).

Changes in the BSFL microbiome could affect greenhouse gas emissions, especially the production of methane, which is exclusively attributed to the Archaea (Enzmann et al., 2018). Therefore, also studies about the archaeal microbiota are of major importance in the context of large-scale insect farming. Methane emission from BSFL farms is generally low but varies between locations (Ermolaev et al., 2019; Mertenat et al., 2019; Parodi et al., 2020). When BSFL are reared on chicken feed mixed with 0.3% rabbit manure, 28.54% of the carbon initially present within the system is lost to the atmosphere in the form of CO_2_ along with negligible amounts of methane (Perednia et al., 2017). Variations in methane emission are likely attributed to differences in the feed source and the gut microbial community, which was not investigated in the studies mentioned above.

Amplicon sequencing has shown that only ∼0.02% of all sequences obtained from BSFL guts belong to archaea, most of them methanogens (Klammsteiner et al., 2020). However, there is no evidence that this small population includes metabolically active residents of the gut. Indeed, we used primers designed to include both Bacteria and Archaea, but none of the sequences we recovered were classified as Archaea. This suggests that BSFL reared on CPC or chicken feed do not produce significant amounts of methane, which is favorable for large-scale insect farming.

### The BSFL Fungal Community

We found that the fungal community of the BSFL gut was dominated by Trichosporonaceae (*Trichosporon asahii*) in both diet groups. The same fungus was also dominant in another study of BSFL reared on chicken feed and present (albeit in lower abundance) in BSFL reared on other diets (Varotto Boccazzi et al., 2017) suggesting this species is part of the core microbiome. Interestingly, the frass of larvae reared on chicken feed exclusively contained two fungal species (*T. asahii* and *Diutina rugosa*) whereas frass from the CPC group contained a diverse fungal community. The high relative abundance of *T. asahii* in gut and frass samples (both diets) and *D. rugosa* in gut samples (both diets) as well as in chicken feed frass, indicates that these yeasts are functionally associated with BSFL and may have a probiotic effect. Mutualistic associations between yeasts and insects are common, especially in the orders Diptera, Hymenoptera and Coleoptera (Vega and Dowd, 2005; Vogel et al., 2017; Madden et al., 2018; Stefanini, 2018). The genus *Diutina* was also found in *Drosophila* spp. (Phaff et al., 1956; Camargo and Phaff, 1957) and ants (Ba and Phillips, 1996). Moreover, some yeasts can bind to chitin (Ishijima et al., 2017) via cell-surface adhesion proteins known as flocculins (Brückner and Mösch, 2012). This may allow the attachment of yeasts to the cuticle in the foregut and hindgut, and to the peritrophic membrane in the midgut, thus promoting stable colonization.

The probiotic effect of *D. rugosa* has been shown to increase body weight and feed conversion ratios in poultry (Wang et al., 2019a, b). *D. rugosa* probably helps to establish a healthy gut and substrate microbiome in BSFL and, if not present naturally in farmed populations, could be applied as a feed supplement in the future. *T. asahii* may also help to establish a healthy microbiome, given its antimicrobial activity against the pathogenic yeasts *Candida glabrata* and *C. lusitaniae* (Varotto Boccazzi et al., 2017). *T. asahii* and *D. rugosa* may also protect BSFL from *Fusarium* species, which are pathogenic to many insects, including the Diptera (Santos et al., 2020). We detected abundant *Fusarium* species in all feed samples but these potential entomopathogens were completely absent from the guts of BSFL reared on chicken feed and scarce in the guts of BSFL reared on CPC. *Trichosporon* spp. may be beneficial for BSFL development and the establishment of a healthy gut microbiome, but their numbers must be controlled in BSFL farms because they are also opportunistic human pathogens that cause infections in immunocompromised patients (Colombo et al., 2011). Similarly, the presence of Cladosporiaceae (all *Cladosporium*) in almost all samples was anticipated because this mold fungus is ubiquitous, but the numbers must be controlled in BSFL farms because these fungi can cause allergic reactions (Bavbek et al., 2006).

Like the bacterial microbiome, the fungal gut microbiome was diet-dependent, as shown by the higher alpha diversity in BSFL reared on CPC, which may reflect the characteristic features of the substrate including complex compounds such as gossypol. The Aspergillaceae (most belonging to the genus *Aspergillus*) were abundant in the guts of larvae reared on CPC but completely absent in the guts of larvae reared on chicken feed, despite their presence in the feed samples. The Aspergillaceae are known for their ability to break down gossypol (Yang et al., 2011, 2012; Mageshwaran et al., 2017; Grewal et al., 2020) suggesting they may metabolize this compound in the BSFL gut. However, the Aspergillaceae also produce aflatoxins and gliotoxins (Bennett and Klich, 2003). Although black soldier flies tolerate high levels of aflatoxins and do not accumulate aflatoxin B1 (Bosch et al., 2017), the potential accumulation of other mycotoxins should be investigated in more detail before using CPC routinely as insect feed. Furthermore, Trichocomaceae and Microascaceae were found in larvae from the CPC diet group but not the chicken feed diet group, suggesting these families may also contribute to the degradation of gossypol.

## Conclusion and Outlook

Here we could show, that BSFL can be reared on CPC as the sole diet for several generations without any problems. The shorter life cycle and higher rates of successful development of black soldier flies reared on CPC might also be beneficial for large-scale farming and colony maintenance. Nevertheless, the growth pause and the lower final weight would be disadvantageous for a large-scale production. BSFL appear to tolerate certain amounts of gossypol and might be capable of detoxifying the substrate by degrading this compound. However, this needs to be confirmed by analytical gossypol quantification of BSFL and frass samples in the future. Furthermore, it needs to be tested if the developmental, physiological and nutritional properties of BSF reared at this small scale can apply for industrial insect farming. Also, variations in nutritional value, gossypol concentration and the overall quality of CPC, depending on the cottonseed variety, manufacturing process and storage conditions must be considered.

Amplicon sequencing of bacterial 16S rRNA genes and fungal ITS sequences revealed a functional core microbiome that may protect BSFL from pathogens, but also highlighted pronounced diet-dependent differences in the microbial community of the BSFL gut and frass. There was little similarity between the bacterial communities of the gut and frass, but more similarity between the corresponding fungal communities, especially those representing the chicken feed diet group. The high relative abundance of yeasts such as *D. rugosa* and *T*. *asahii* suggests these fungi might play a beneficial role during the rearing of BSFL.

The high relative abundance of the Aspergillaceae and *Actinomycetaceae* only in larvae reared on CPC revealed diet-dependent differences in the gut microbiome possibly indicating specific dietary adaptations. The Aspergillaceae are known for their ability to metabolize gossypol and we detected them only in larvae reared on CPC, suggesting they may also degrade gossypol within the BSFL gut. Our ITS data may reveal other gossypol-metabolizing fungi that could be added as feed supplements in large-scale insect farms using CPC as a major substrate. Bacterial gossypol degradation is still generally sparsely investigated and it is unclear whether it occurs in the BSFL. This can only be confirmed by establishing pure cultures and testing them directly for their metabolic activity *in vitro* accompanied by comparative genomics. Our amplicon data provide essential information for our on-going isolation and screening of potentially gossypol-degrading bacteria and other beneficial microorganisms which might be exploited for industrial applications in the future.

## Data Availability Statement

The datasets presented in this study can be found in online repositories. The amplicon sequencing data can be accessed at NCBI (https://www.ncbi.nlm.nih.gov) under the BioProject PRJNA674583, which includes the 16S rRNA gene and ITS sequences with the following accession numbers: SRX9457342–SRX9457395.

## Author Contributions

DT, SH, KB, PK, and AV designed the experiments. DT performed molecular work, processed the samples, collected developmental and physiological data, evaluated, visualized and discussed the data, and wrote the manuscript. SH analyzed Illumina sequences, evaluated, discussed and visualized the data, and wrote the manuscript. PK collected developmental, physiological and nutritional data, analyzed and discussed the data. KB, PH, and AV evaluated and discussed the data and edited the manuscript. All authors contributed to the article and approved the submitted version.

## Conflict of Interest

The authors declare that the research was conducted in the absence of any commercial or financial relationships that could be construed as a potential conflict of interest.
