# Supplementary material for: Cottonseed Press Cake as a Potential Diet for Industrially Farmed Black Soldier Fly Larvae Triggers Adaptations of Their Bacterial and Fungal Gut Microbiota
Source: Front Microbiol. 2021 Mar 29;12:634503. doi: 10.3389/fmicb.2021.634503 (PMC8039154; doi:10.3389/fmicb.2021.634503)
Supplement: Supplementary file 3 [file Data_Sheet_1.docx]

Supplementary Material

# Supplementary Tables and Figures

## Supplementary Tables

Supplementary Table 1. Temperature of the substrate with of black soldier fly larvae (BSFL) reared on chicken feed (CF) and cottonseed press cake (CPC). Values are means of three replicate boxes ± standard deviation.

| **CF** | | **CPC** | |  |
| --- | --- | --- | --- | --- |
| Day | Temperature °C | Day | Temperature °C |  |
| 0 | ND | 0 | ND |  |
| 4 | 27.0 ± 0.12 | 4.0 | ND |  |
| 6.1 | 27.6 ± 0.00 | 6.0 | ND |  |
| 7.9 | 28.8 ± 1.17 | 8.0 | 28.8 ± 0.14 |  |
| 9.9 | 32.5 ± 1.67 | 10.0 | 28.2 ± 0.35 |  |
| 12.0 | 32.7 ± 1.55 | 12.0 | 29.1 ± 0.28 |  |
| 15.0 | 33.2 ± 0.40 | 13.9 | 30.0 ± 0.87 |  |
| 16.9 | 31.8 ± 0.40 | 15.9 | 29.7 ± 0.44 |  |
| 18.7 | 32.2 ± 0.36 | 17.9 | 30.6 ± 0.46 |  |
| 21.0 | 31.0 ± 1.50 | 20.2 | 30.2 ± 0.81 |  |
| 21-day average | 30.8 ± 2.36 | 20-day average | 29.5 ± 0.84 |  |

## Supplementary Figures


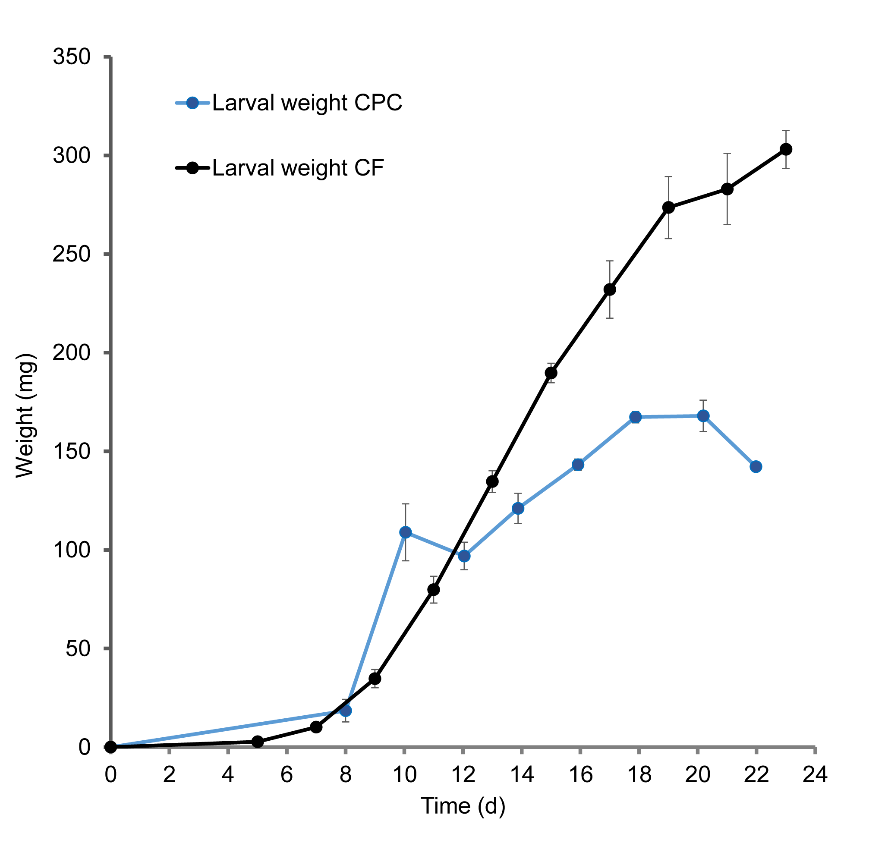


**Supplementary Figure 1**. Growth curve of BSFL reared on chicken feed (CF) and cottonseed press cake (CPC). Growth experiment shown in Figure 1 was repeated here in order to evaluate reproducibility of the results. The average body weight per BSFL is shown. Symbols are means (+ SEM) of measurements with three replicates.

**
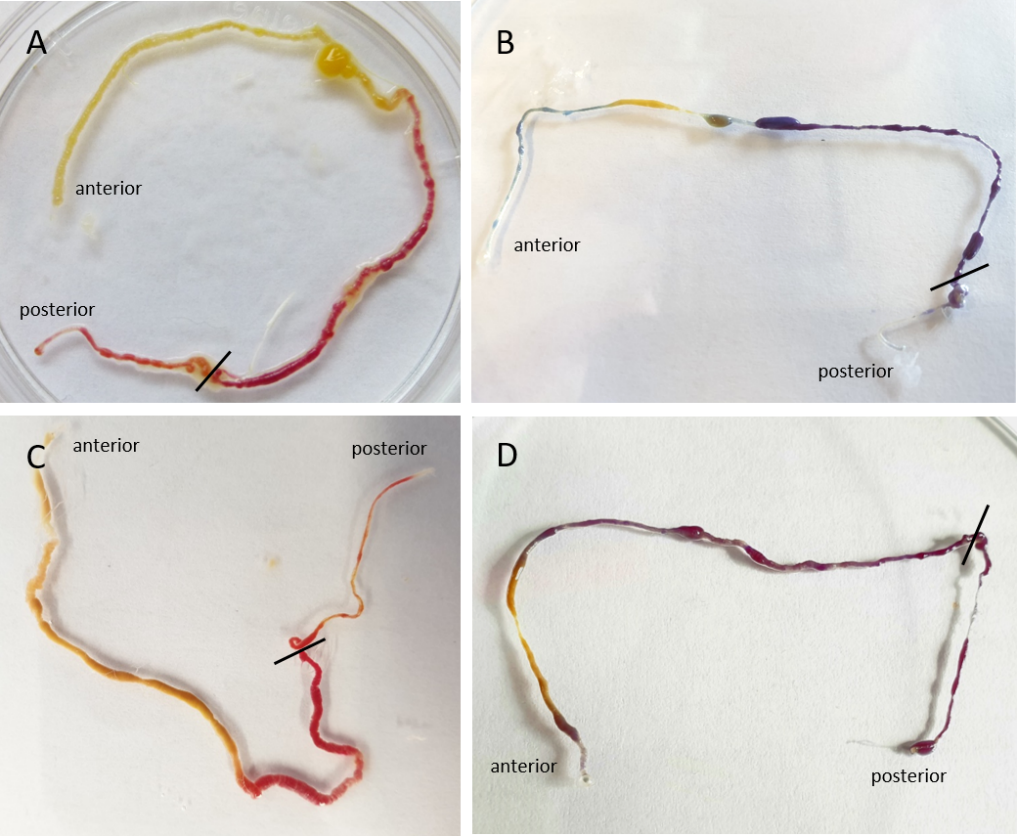
**

**Supplementary Figure 2.** Guts of BSFL reared on chicken feed (CF) or cottonseed press cake (CPC) after feeding with 0.2% phenol red or 0.2% bromophenol blue, indicating the pH differences along the gut lumen (midgut and hindgut). **(A)** Gut of BSFL reared on CF colored with phenol red; **(B)** Gut of BSFL reared on CF colored with bromophenol blue; **(C)** Gut of BSFL reared on CPC colored with phenol red; **(D)** Gut of BSFL reared on CPC colored with bromophenol blue. The transition from midgut to hindgut is indicated by a parting line.


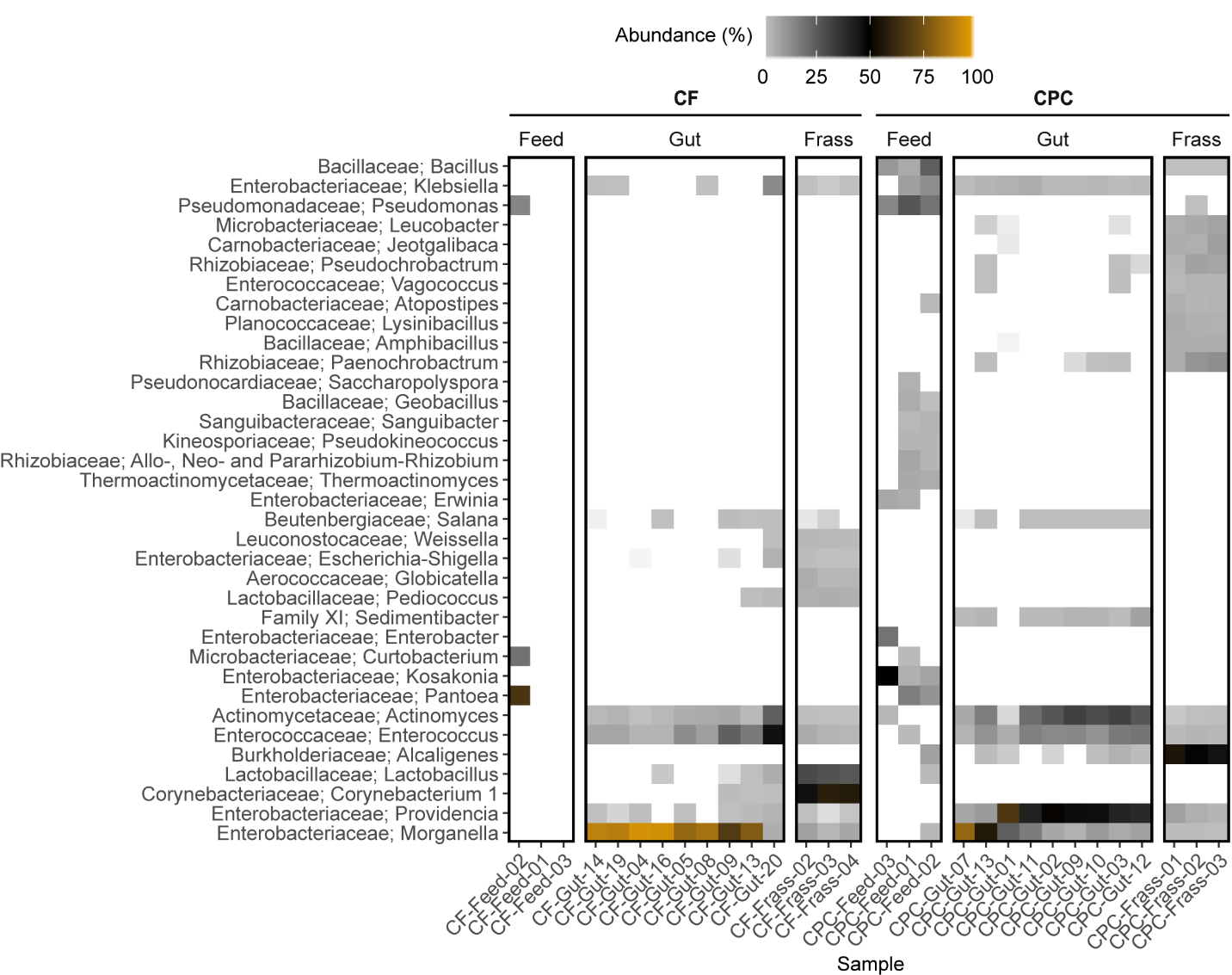


**Supplementary Figure 3.** Heat map showing the genus-level composition of the bacterial community of feed, BSFL guts and BSFL frass in the chicken feed (CF) and cottonseed press cake (CPC) diet groups based on 16S rRNA gene amplicon sequencing. The relative abundances of amplicon sequence variant (ASV) counts are collapsed to the genus level for all replicates. Undefined genera are not shown. Percentages of relative abundances are displayed by coloring from light gray over black to orange representing the highest abundance. Genera depicted in white were not detected in the sample. Only the top 35 genera are shown, measured by the mean counts per genus level for all samples.

**
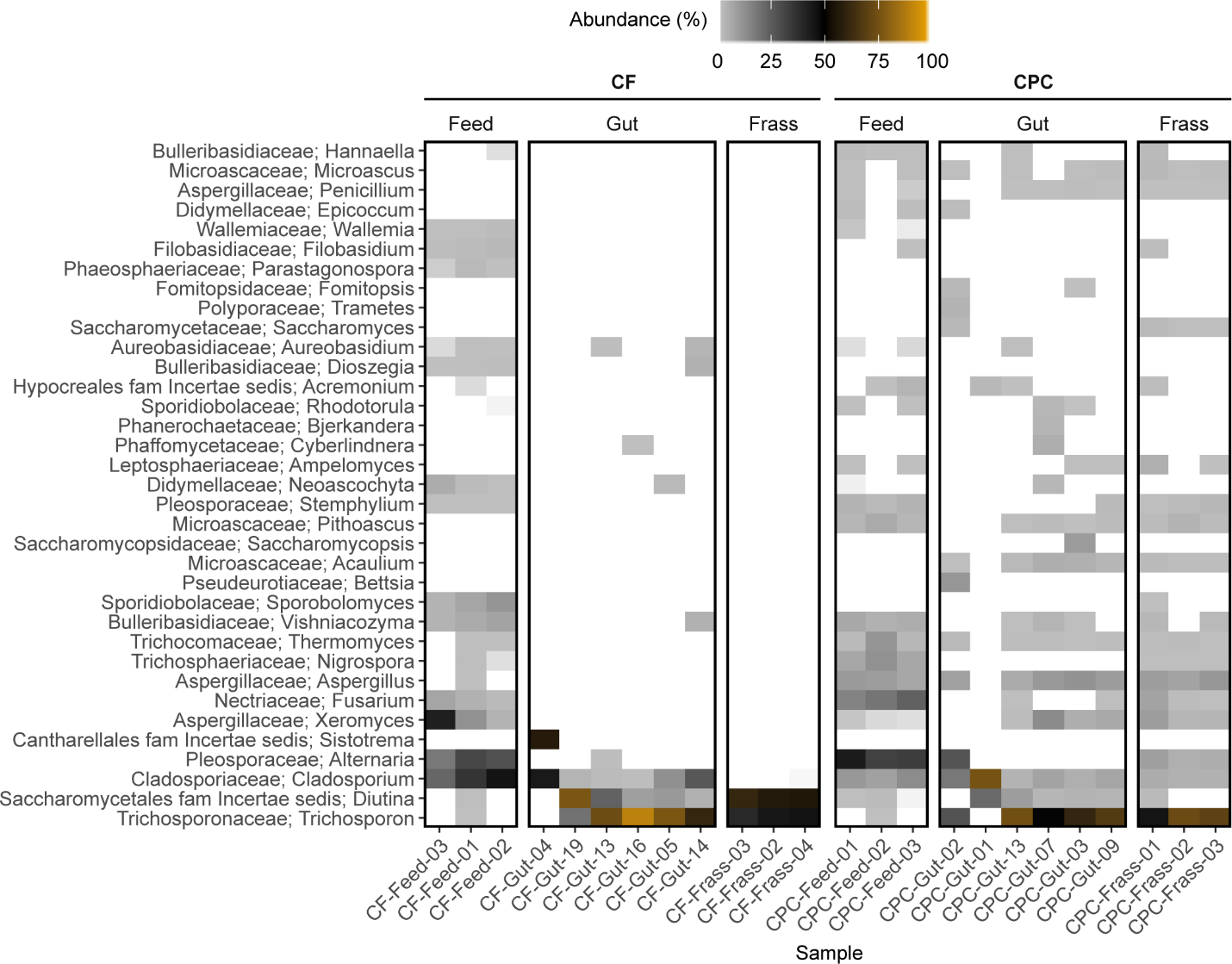
**

**Supplementary Figure 4**. Heat map showing the genus-level composition of the fungal community of feed, BSFL guts and BSFL frass in the chicken feed (CF) and cottonseed press cake (CPC) diet groups based on ITS amplicon sequencing. The relative abundances of amplicon sequence variant (ASV) counts are collapsed to the genus level for all replicates. Undefined genera are not shown. Percentages of relative abundances are displayed by coloring from light gray over black to orange representing the highest abundance. Genera depicted in white were not detected in the sample. Only the top 35 genera are shown, measured by the mean counts per genus level for all samples.
